# Radiation Therapy for Maxillary Sinus Epithelioid Hemangioendothelioma: A Case Report and Literature Review

**DOI:** 10.7759/cureus.91590

**Published:** 2025-09-04

**Authors:** Salma Ichou, Amine Lachgar, Ihsane Skitioui, Karima Nouni, Hanane Elkacemi, Tayeb Kebdani, Khalid Hassouni

**Affiliations:** 1 Radiation Oncology, National Institute of Oncology, Mohammed V University, Faculty of Medicine, Rabat, MAR

**Keywords:** epithelioid hemangioendothelioma (ehe), hemangioendothelioma, intensity-modulated radiotherapy (imrt), maxillary sinus, relapse, sarcoma

## Abstract

Epithelioid hemangioendothelioma (EHE) is a rare vascular tumor arising from endothelial cells, with potential involvement of various anatomical sites. We report the case of a 37-year-old woman presenting with a locally advanced recurrence of maxillary sinus EHE. Due to the tumor's extent and anatomical constraints, complete surgical resection was not feasible. The patient was treated with intensity-modulated radiotherapy (IMRT), achieving a favorable clinical and radiological response.

This case highlights the potential of IMRT as an effective therapeutic option for unresectable, locally advanced EHE, with acceptable acute toxicity. Systemic therapy may also be considered in aggressive forms to improve symptom control and progression-free survival (PFS). However, the optimal sequence between systemic treatment and radiotherapy remains unclear when surgery is not an option.

## Introduction

Epithelioid hemangioendothelioma (EHE) is an exceptionally rare vascular neoplasm of endothelial origin, with an estimated incidence of approximately one case per million individuals [1]. While it can arise in diverse anatomical locations, the liver, lungs, and bones are the most commonly affected organs. In contrast, head and neck involvement, and particularly localization to the maxillary sinus, is exceedingly uncommon, with only a handful of cases described to date [2,3].

The clinical course of EHE is highly heterogeneous, ranging from indolent, slowly progressive tumors to aggressive forms with multifocal spread and distant metastases [4]. This variability, coupled with its rarity, poses substantial diagnostic and therapeutic challenges. 

Given the absence of standardized treatment guidelines, therapeutic strategies for EHE vary widely and may include surgical resection, systemic therapies, radiotherapy, or even surveillance in selected cases. For tumors arising in anatomically complex regions such as the maxillary sinus, surgery may be technically challenging or associated with significant morbidity.

In this context, radiotherapy emerges as a particularly important modality, offering the potential for effective local control while preserving function. Advances in radiotherapy techniques, including intensity-modulated radiation therapy (IMRT), allow for highly conformal dose delivery, maximizing tumor control while minimizing toxicity to adjacent critical structures in the head and neck.

Because of these uncertainties, the documentation and analysis of rare presentations such as maxillary sinus EHE are essential. In this context, we present a case of maxillary sinus EHE managed with radiotherapy, accompanied by a review of the literature, in order to provide a broader perspective on clinical presentation, diagnostic challenges, and therapeutic strategies for this rare entity.

## Case presentation

**Figure 1:**
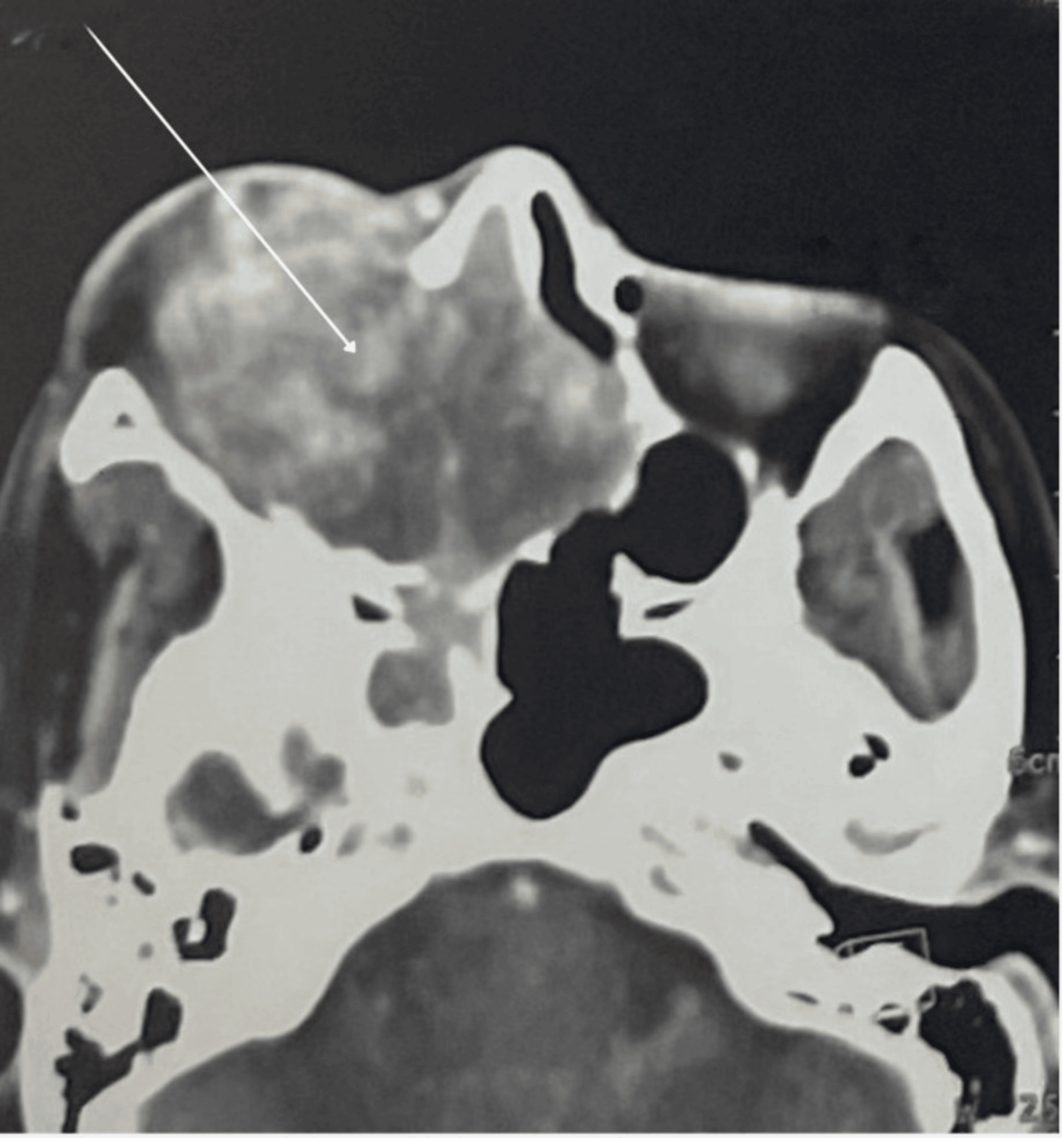
Right maxillary process and nasal cavity was seen protruding into the nasal septum (before the resection)

We report the clinical course and pathologic features of a 37-year-old woman with no significant comorbidities who presented with epistaxis and progressive left jugular swelling over seven months prior to her initial surgery. Physical examination revealed swelling and deformity of the right hemiface. Radiographic imaging showed a large, soft tissue lesion filling the maxillary sinus with significant bony erosion and expansion (Figure 1).

The patient was diagnosed with EHE and underwent endoscopic resection. Three months later, follow-up cervico-facial CT imaging revealed a maxillary soft tissue mass suggestive of tumor recurrence.

**Figure 2:**
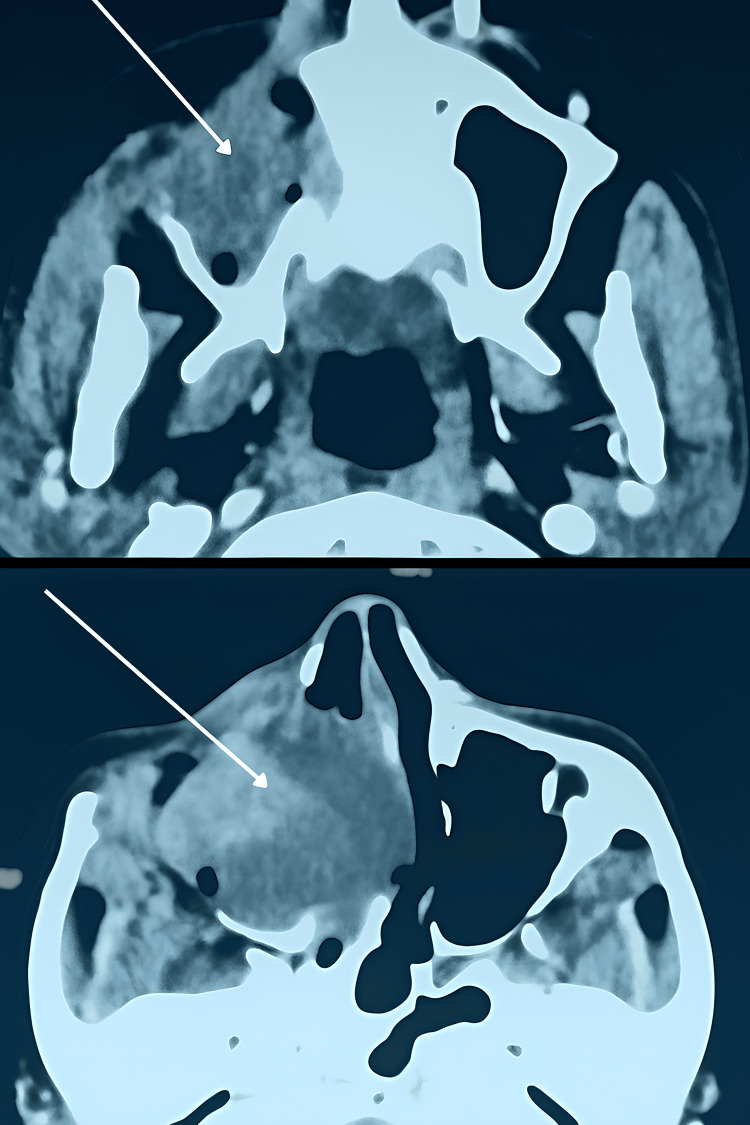
The CT showed a heterogeneous tumor measuring 46x40x30 mm containing an area of necrosis and extending across the nasal septum toward the contralateral side (three months after the resection).

The lesion was centered in the right maxillary sinus and ipsilateral nasal cavity, with extension across the nasal septum toward the contralateral side. The mass exhibited irregular contours, contained areas of necrosis, and showed heterogeneous enhancement following contrast injection (contrast agent), with approximate dimensions of 46 × 40 × 30 mm (Figure 2).

**Figure 3:**
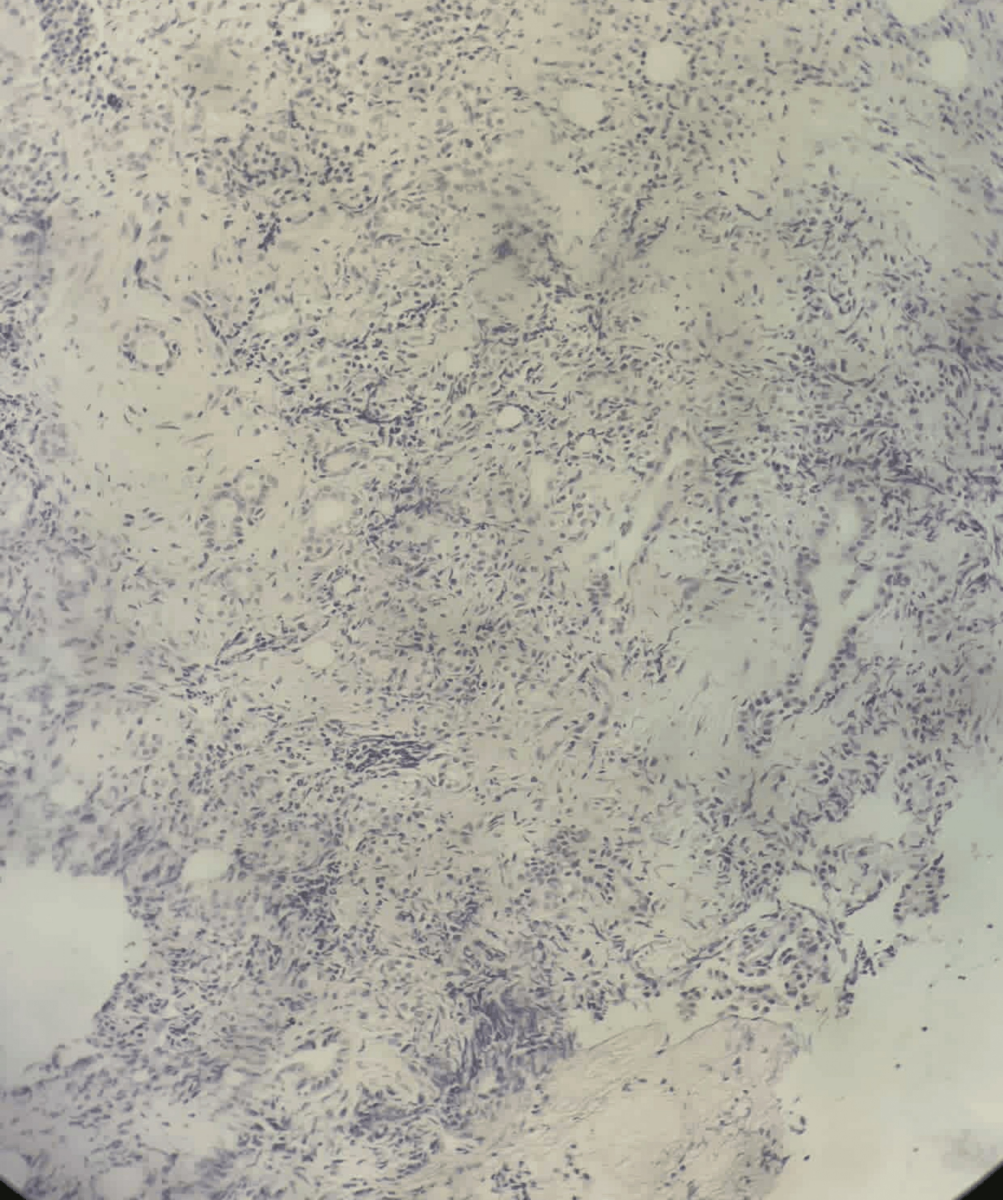
A histological section stained with H&E showed a proliferation of tumor endothelial cells with epithelioid morphology, arranged in clusters and cords within a myxoid stroma. The cells exhibited abundant eosinophilic cytoplasm and rounded nuclei. The presence of rudimentary vascular lumina or intracytoplasmic vacuoles was suggestive of vascular differentiation.

Histological evaluation of the lesion demonstrated a spindle cell proliferation of variable density, ranging from minimal to moderate, admixed with a prominent inflammatory infiltrate composed predominantly of plasma cells and lymphocytes, with scattered eosinophils and mast cells. The spindle cells exhibited eosinophilic cytoplasm with tapered or band-like extensions. Their nuclei are either monomorphic with fine chromatin or show anisokaryosis with vesicular nucleoli. The background stroma was myxoid and highly vascularized, with the presence of fibrinocruor thrombi. Mitotic activity was low, with only one mitosis observed per 10 high-power fields at ×40 magnification. Additional findings included storiform fibrous remodeling, surface ulceration, and infiltration into surrounding striated muscle and adipose tissue (Figure 3).

Immunohistochemical (IHC) analysis revealed that plasma cells were positive for both kappa and lambda light chains. IgG staining was positive in many plasma cells, while only approximately 1% stained positive for IgG4. The patient’s serum IgG4 level was within normal limits at 0.27 g/L. ERG immunostaining was positive in numerous spindle cells. FOSB and CAMTA1 antibodies were not available for testing in this case. Molecular testing using next-generation sequencing (NGS) did not reveal any ALK gene rearrangement. Upon slide review, areas containing small vascular lumina filled with red blood cells and lined by one or more tumor cells were also identified.

Given the tumor’s locally aggressive behavior and its close proximity to the right optic nerve, surgical resection was deemed unfeasible. After a multidisciplinary discussion, the decision was made to proceed with exclusive radiation therapy without chemotherapy. The patient underwent IMRT, receiving a total dose of 66 Gy to the primary tumor volume in 33 fractions of 2.0 Gy each and 56 Gy to the cervical lymph node chains in 28 fractions of 2.0 Gy each.

**Figure 4:**
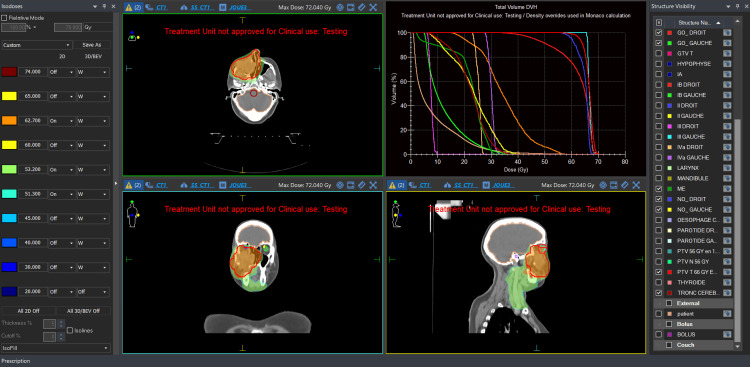
Radiation plan for the tumor (66 Gy)

**Figure 5:**
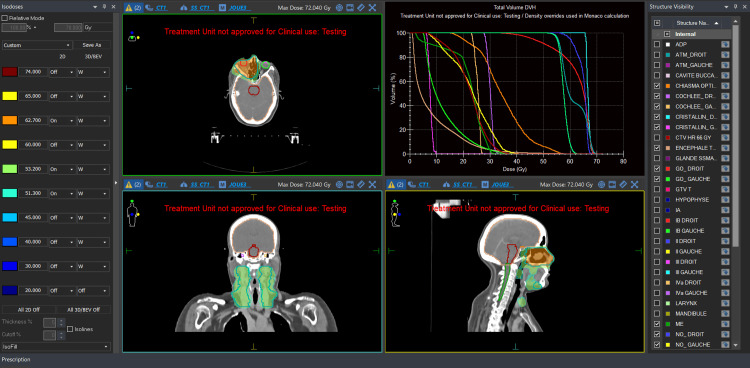
Radiation plan for the neck (56 Gy)

The gross tumor volume (GTV) was defined based on CT findings, encompassing all visible tumor tissue. Clinical target volumes (CTVs) were generated by adding a 5 mm margin to the GTV. Specifically, CTV-T included the GTV plus 5 mm, with particular attention to minimizing radiation exposure to the contralateral optic nerve (Figure 4). CTV-N encompassed bilateral cervical lymph node levels Ia, II, III, and IVa (Figure 5).

Planning target volumes (PTVs) for both the primary tumor and the neck were created by expanding each CTV by an additional 5 mm margin. Daily image-guided radiotherapy using cone-beam CT (CBCT) was employed to ensure precise targeting.

The patient completed the treatment course with good overall tolerance. Reported acute toxicities included grade I radiation dermatitis and persistent epiphora (tearing) of the right eye, without any high-grade complications.

**Figure 6:**
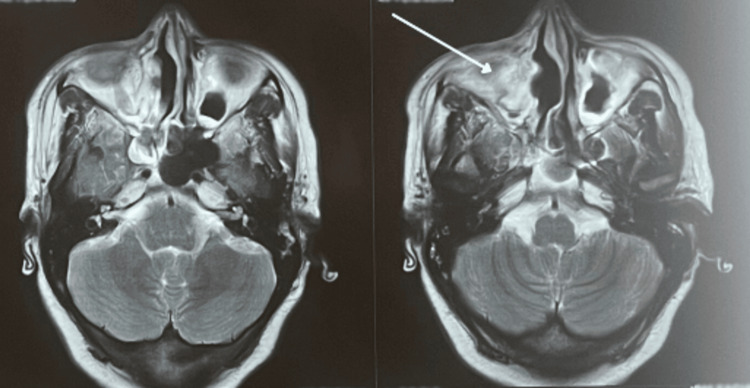
Facial MRI revealed a tumoral process centered on the right maxillary sinus, with locoregional infiltration, in relation to a residual focus.

The patient underwent regular clinical evaluations and serial MRIs every three months, the most recent being 18 months after radiotherapy, which demonstrated significant clinical improvement, including resolution of epistaxis and right hemifacial deformity. Imaging showed marked regression of the lesion, now measuring 25×26×27 mm, with only a small residual focus remaining (Figure 6).

## Discussion

EHE is an ultra-rare vascular sarcoma, first described by Weiss and Enzinger in 1982. EHE has an annual incidence of approximately 0.038 per 100,000 population and a prevalence of less than one in 1,000,000, with a slight female predominance [5]. The rarity of this neoplasm, the unusual anatomic location, and the non-specific symptoms present diagnostic and management challenges [6].

Epithelioid vascular tumors encompass a spectrum of benign and malignant tumors. It can arise anywhere in the body and have various presentations. EHE itself has an intermediate malignant behavior pattern, though cases with indolent behavior have been reported. Among head and neck sites, the submandibular region is the most frequently involved [7].

In our patient, we were faced with aggressive and rapidly progressive symptoms. Most cases of EHE are associated with low mortality, but some show metastasis and can result in the mortality of the patient [8].

The tumor's characteristics are largely consistent across different organs, although the clinical presentation and associated signs and symptoms vary, and the most common symptoms are pain (40%), a palpable mass (6% to 24%), and weight loss (9%), which is similar to our case [9].

Microscopically, EHE is typically composed of epithelioid cells arranged in strands, cords, or solid nests, characterized by a glassy eosinophilic cytoplasm often containing cytoplasmic vacuoles. These cells are embedded in a distinctive myxohyaline stroma. It has a low mitotic activity and mild nuclear atypia [10].

At the molecular level, specific genetic alterations have been associated with this disease. Notably, recent studies have identified a recurrent WWTR1-CAMTA1 gene fusion in EHE, resulting from a t(1;3)(p36;q25) translocation [9,11].

According to the 2021 European Society for Medical Oncology (ESMO) guidelines, EHE is molecularly characterized in most cases by WWTR1-CAMTA1 (≈90%) or, less commonly, YAP1-TFE3 (≈10%) gene fusions, which promote tumorigenesis through dysregulation of endothelial proliferation and Hippo pathway signaling [12].

Rare WWTR1 translocations involving alternative fusion partners have also been described. The detection of these molecular rearrangements is necessary to confirm the diagnosis and exclude histologic mimics such as angiosarcoma or epithelioid hemangioma; IHC or molecular testing for WWTR1-CAMTA1 and/or YAP1-TFE3 is strongly recommended. But to date, none of the translocations identified in EHE are targetable by available drug therapies. Upon confirmation of this histology, whole-body imaging, including CT or MRI, or both, should be carried out to detect trunk or limb pathology. 

Treatment of EHE patients depends upon the location and spread of the disease. Surgery is the treatment of choice for localized, unifocal EHE. It must be performed in referral centers. Surgery has to be a complete resection of EHE with a microscopic negative margin (R0) to expect a cure rate of 70% to 80% [13].

In the case of a soft tissue EHE, the tumor resection has to be “en bloc”. If a macroscopic resection is not feasible or if significant morbidity is anticipated, surgery may be replaced by radiotherapy [14].

Currently, there is no evidence on the use of systemic therapy in neoadjuvant or adjuvant treatment. While conventional systemic chemotherapy is a cornerstone in the treatment of soft tissue sarcomas (STS), its role in EHE is far less defined. It appears to have limited efficacy in EHE and offers minimal benefit in most cases. As such, systemic treatment should be reserved for patients with aggressive or rapidly progressive disease, particularly those whose tumors exhibit features akin to high-grade STS. mTOR inhibitors have demonstrated the highest clinical activity, with median progression-free survival (PFS) and overall survival (OS) of around one and two years, respectively, and prolonged PFS in approximately 10% of patients. The panel considers them the preferred option for advanced, moderately progressive disease.

A phase 2 single-arm trial evaluated the efficacy and safety of trametinib, a MEK inhibitor, in patients with locally advanced or metastatic EHE. The rationale was based on preclinical evidence suggesting activation of the MAPK/MEK pathway, particularly related to the WWTR1-CAMTA1 fusion seen in EHE. The study showed that trametinib was associated with a reduction in EHE-related pain and a median PFS of more than six months, providing palliative benefit. However, the trial did not meet its primary endpoint of objective response rate (ORR) [15].

EHE of the maxillary sinus is extremely rare, with only a few cases reported in the literature. Most reported cases have been managed surgically, and the role of radiotherapy remains poorly defined. In our case, IMRT was chosen as the primary local treatment due to the tumor’s proximity to critical structures and the inability to achieve complete surgical resection. The highly conformal dose distribution allowed effective tumor control while minimizing exposure to surrounding tissues, suggesting that IMRT may represent a valuable option in anatomically challenging locations.

In addition, proton therapy may offer a promising alternative in cases where conventional surgery or radiotherapy is limited by proximity to critical structures.

A review of the literature on the primary EHE of the maxillary sinus was done. The search was performed in PubMed and Cureus with the terms “epithelioid hemangioendothelioma” and “maxillary sinus.” All the data are compiled in Table 1.

**Table 1:** Summary of reports of epithelioid hemangioendothelioma of the maxillary sinus

Authors (year)	Age (years)	Sex	Clinical stage	Treatement	Adjuvant treatment	Evolution
Avadhani et al., 2016 [16]	72	Female	Localised	Surgery		No recurrence
Wong et al., 2016 [17]	73	Male	Locally advanced	Surgery		-
Chaouki et al., 2019 [18]	18	Male	Locally advanced	Radiation therapy	55 Gy of intensity-modulated radiation therapy + chemotherapy (cisplatine 40mg/m2)	50% regression after 18 months of follow-up
Talebzadeh et al., 2023 [19]	66	Female	Locally advanced	Surgery		-
Current case	37	Female	Locally advanced	Surgery + Radiation therapy	66 Gy of intensity-modulated radiation therapy without chemotherapy	No recurrence 18 months post treatment

## Conclusions

EHE of the maxillary sinus is a rare vascular tumor with intermediate malignant potential, often challenging to diagnose. This case underscores the critical importance of integrating clinicopathologic, radiologic, and molecular findings for accurate diagnosis and risk stratification. In the absence of standardized treatment guidelines, therapeutic decisions should be tailored based on tumor resectability, anatomical limitations, and biological behavior.

Our experience highlights the promising role of IMRT as an effective option for locally advanced, unresectable recurrences, with manageable acute toxicity. Systemic therapy also represents a valuable approach for locally aggressive disease, contributing to symptom relief and PFS. The optimal sequencing of systemic treatment and radiotherapy remains to be clarified when surgery is not feasible. Given the rarity of maxillary sinus EHE and the scarcity of long-term outcomes, multicenter collaborations and comprehensive molecular profiling are crucial to refine prognostic models and develop evidence-based management strategies.
